# A Case of De Novo Anterior Condylar Dural Arteriovenous Fistula Long after Curative Transvenous Embolization of Contralateral Anterior Condylar Arteriovenous Fistula

**DOI:** 10.1155/2016/6974526

**Published:** 2016-10-18

**Authors:** Shinya Hagiwara, Takeshi Miyazaki, Masahiro Tsuji, Mizuki Kambara, Tsutomu Yoshikane, Hidemasa Nagai, Yasuhiko Akiyama

**Affiliations:** Department of Neurosurgery, Shimane University Faculty of Medicine, Shimane, Japan

## Abstract

We report on a 55-year-old man who developed a de novo DAVF in left ACC 5 years after curative transvenous embolization for DAVF in right ACC. Angiography revealed that the de novo lesion demonstrated more aggressive arteriovenous shunt flow than the initial lesion. Successful transvenous embolization was performed for also the second lesion. The authors describe the possible pathophysiological mechanisms and management strategies for this rare occurrence.

## 1. Introduction

Cranial dural arteriovenous fistula (DAVF) is abnormal arteriovenous shunts. It occurs anywhere in cranial sinus, commonly in cavernous and transverse sinus, and rarely in anterior condylar confluence (ACC). DAVF is treated with disconnection of the venous drainage system via endovascular intervention or surgical procedures [1]. It is rare that a second DAVF occurs in a remote area after resolution of other located first lesions [2–10]. Most of them develop in the region of downstream of the venous pathway where the first lesion located.

We report a very rare case of a de novo ACC DAVF in the contralateral ACC 3 years after unilateral ACC DAVF, treated by transvenous embolization.

## 2. Case Presentation

**Figure 1:**
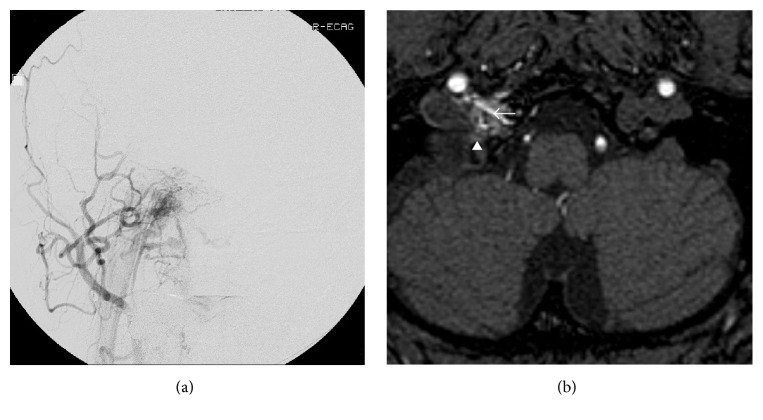
Right external carotid angiogram depicting dural arteriovenous fistula. It is fed by ascending pharyngeal artery and it drains to the vertebral venous plexus (a). Axial source image of time-of-flight MRA shows hyperintense thin vessel penetrating hypoglossal canal (arrow) and arterialized venous pouch of right anterior condylar confluence (arrowhead).

A 55-year-old man was admitted to our hospital with sudden pulsatile headache and pulse-synchronous tinnitus on the left occipital. Bruit was audible mainly on his left retroauricular area. He had past history of right ACC DAVF at 50 years of age (Figure 1). Its clinical manifestation was right-sided tinnitus and it was similar to the present symptoms. His right ACC DAVF was treated with transvenous coil embolization in another hospital and its obliteration was angiographically confirmed. He had resolved clinical symptoms and stopped visiting the hospital 1 year after the treatment.

**Figure 2:**
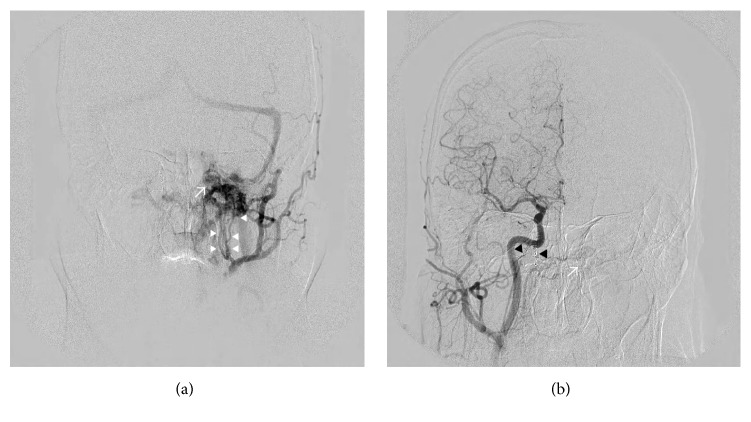
(a) Left external carotid angiogram demonstrates dural arteriovenous fistula around left jugular valve. Dural arteriovenous fistula is mainly fed by meningeal branches of the left ascending pharyngeal artery (arrowhead) and drained to left internal jugular vein, bilateral vertebral venous plexus through the left anterior condylar vein. Left cavernous sinus and transverse-sigmoid sinus are refluxed. Anterior condylar confluence is depicted as expanded venous pouch (arrow). (b) Right common carotid angiogram demonstrated obliteration of dural arteriovenous fistula in the right anterior condyle confluence. Coil mass inserted five years ago is depicted (arrowhead). Right ascending pharyngeal artery feeds dural arteriovenous fistula in the contralateral anterior condylar confluence.

**Figure 3:**
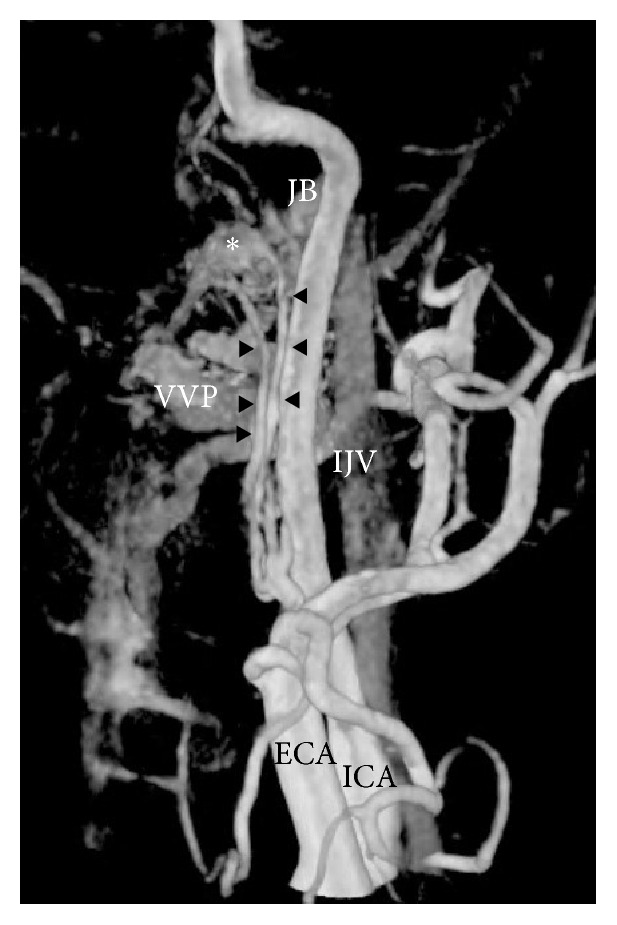
Three-dimensional digital subtraction angiography revealed angioarchitecture surrounding the anterior condyle confluence dural arteriovenous fistula. Meningeal branches of ascending pharyngeal artery feed to anterior condyle confluence forming venous pouch (*∗*). Jugular valve (JV), internal jugular vein (IJV), and vertebral artery venous plexus (VAVP) formed venous drainage.

**Figure 4:**
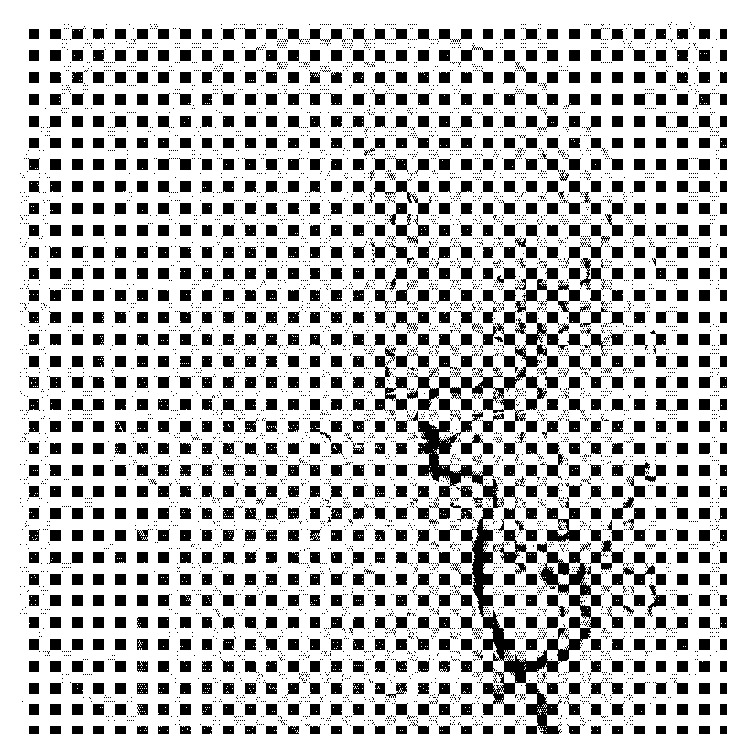
Left common carotid angiogram demonstrates complete obliteration of anterior condylar confluence dural arteriovenous fistula.

**Figure 5:**
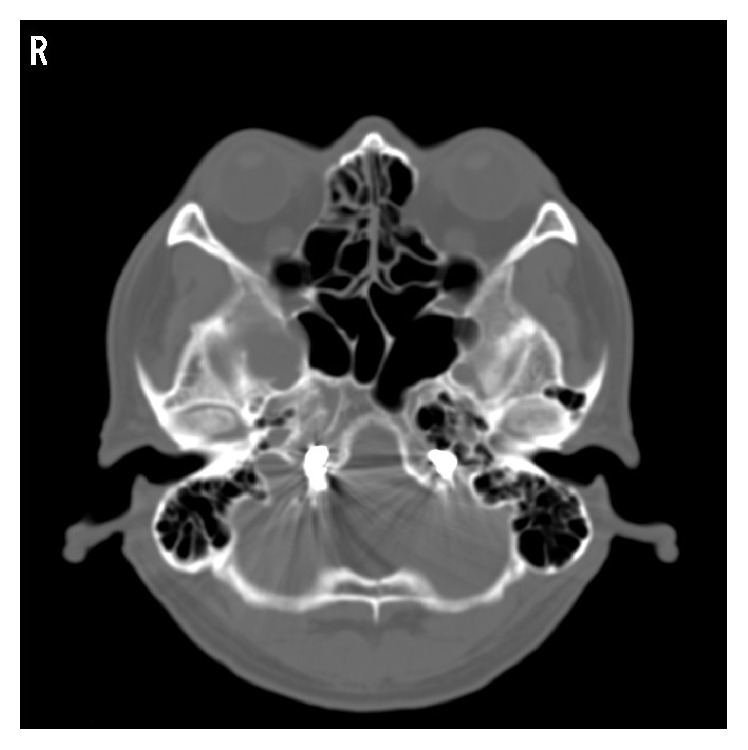
CT after treatment demonstrates packing coil seated in right ACC to hypoglossal canal (treated 5 years ago) and in left ACC.

The diagnostic angiography in our hospital revealed a high flow DAVF close to left jugular foramen, and it was mainly fed by meningeal branch of bilateral ascending pharyngeal arteries. It was not only drained to left internal jugular vein and vertebral artery venous plexus, but also refluxed to contralateral transverse-sigmoid sinus and to cavernous sinus via inferior petrosal sinus. Those findings suggest this lesion to have high volume arteriovenous shunt flow (Figure 2(a)). The shunt point could not be identified by routine angiography because blood flow to the lesion was very high. The right ACC DAVF treated five years earlier was not depicted on the four vessels' study (Figure 2(b)). The shunt point was evaluated by angioarchitecture demonstrated by three-dimensional angiography and by time-of-flight magnetic resonance angiography (MRA). Shunt point was identified on left ACC (Figure 3), and transvenous coil embolization was planned. Under general anesthesia, a 6 Fr guiding catheter was placed adjacent to the jugular bulb via right femoral vein, and a microcatheter was advanced into the left ACC. Superselective angiography of left ascending pharyngeal artery confirmed that the microcatheter was accurately placed in ACC that is forming venous pouch, and then coil packing was carried out. Excess coil packing in hypoglossal canal results in hypoglossal nerve palsy; therefore, coil embolization was finished with angiography feeble of venous drainage. The patient's symptoms resolved just after the treatment. Follow-up angiography at one month after the embolization revealed complete obliteration of this de novo ACC DAVF (Figure 4), and CT after treatment demonstrates packing coil seated in right ACC to hypoglossal canal (treated 5 years ago) and in left ACC (Figure 5).

## 3. Discussion

A DAVF close to the jugular foramen has been referred to as various entities including the DAVF involving the marginal sinus, hypoglossal DAVF [11], DAVF of the anterior condylar vein within the hypoglossal canal [12], and jugular foramen DAVF [13]. The development of high resolution dimensional techniques for angiography has allowed identification of the shunt point of these DAVFs in small venous complex close to the hypoglossal canal. This complex is referred to as the ACC DAVF. ACC DAVF has its shunt near the petrous bone and patients feel severe tinnitus; however, definitive diagnosis tends to be delayed. Pulsatile compression of the enlarged anterior condylar veins occasionally causes hypoglossal nerve palsy or involuntary movement of the tongue [14].

The present case is the first report of de novo ACC DAVF that occurred after curative treatment of contralateral ACC DAVF. DAVF is generally accepted to be an acquired vascular disease in adults. Although its exact pathogenesis is unclear, it is considered that venous hypertension due to sinus thrombosis enlarges the physiological minute arteriovenous connection which normally presents within the dura matter and results in occurrence of abnormal dural arteriovenous shunt [15]. Animal model and surgical specimens also postulate that sinus thrombosis is the key to develop DAVF [16, 17]. In some cases, multiple lesions are simultaneously found at the time of diagnosis and this is generally called multiple DAVFs. Recent studies revealed that these synchronous multiple lesions are found in 7~8% of intracranial DAVFs [3, 18]. On the other hand, second DAVF development in another sinus after resolution of the first lesion is rare. The cases with such second DAVF can be called metachronous multiple DAVFs. To our knowledge, thirteen cases have been reported [2–10]. Interestingly, most of these second lesions in metachronous multiple DAVF, twelve among the thirteen lesions, had developed downstream of the same venous pathway where the first lesion located. This phenomenon might be also explained by above described theory that venous hypertension in morbid sinus derives DAVF occurrence. The second de novo lesion developed within one year after the obliteration of the first lesion (range from 4 to 92 months). The second DAVF on the ipsilateral side may explain development from persisting venous hypertension after the endovascular shunt point disconnection. There are two cases of reported incidence of de novo DAVF developed on the contralateral side of the initial location including our case. Kurl et al. reported a case of right transverse-sigmoid sinus DAVF that developed twenty-three months after the transarterial embolization with N-butyl cyanoacrylate (NBCA) [6]. In this case, they did not detect signs of thrombosis or deformation on sinuses. Likewise, we did not detect thrombosis or stenosis in any sinus in our case. Although only few cases of de novo lesion on the contralateral side are reported, the interval between the first lesion and the second lesion appears to be longer than ipsilateral occurrence. Not only venous hypertension, but also unknown genetic factor may associate with developing the DAVFs.

Ha et al. studied clinical and angiographic characteristics of multiple DAVF. They found that both synchronous and metachronous multiple DAVFs have aggressive angiographic and clinical symptoms, and they concluded that aggressive management is necessary for these cases with multiple lesions [3]. The present case also demonstrates larger volume of shunt flow and higher degree venous reflux than initial lesion. Secondly developed DAVF may need the aggressive treatment even wherever it occurred. It will be important to find out the second lesion to avoid the poor clinical prognosis of the patient. Recently, MRI including time-resolved MRA imaging is recommend as one of the useful diagnostic tools [19]; however, it is yet unclear what type of patient needs longer follow-up and for how long should the patients be observed after the treatment of DAVF. These still remain as clinical research subject to clarify the pathophysiology of cranial DAVF.

## 4. Conclusion

We reported a rare case of metachronous bilateral ACC DAVF. Although second DAVF development is rare, it may manifest aggressive angiographic and clinical symptoms. Accurate diagnosis in early stage is important, and aggressive management will be mandatory.

## Abbreviations

DAVF: Dural arteriovenous fistula
ACC: Anterior condylar confluence
MRA: Magnetic resonance angiography
NBCA: N-Butyl cyanoacrylate.
